# Development of Vitamin C-Loaded Electrospun Nanofibers of Mixture of Polysaccharides of Pullulan/Xanthan Gum for Fast Dissolving Oral Film Applications

**DOI:** 10.3390/ma17040861

**Published:** 2024-02-12

**Authors:** En Cheng, Zhanhui Geng, Lubing Xiang, Xiaoying Zhao, Aimin Xiang, Huafeng Tian

**Affiliations:** 1 School of Light Industry Science and Engineering, Beijing Technology and Business University, Beijing 100048, China; ench456@163.com (E.C.); xlbingice@163.com (L.X.); xaming@th.btbu.edu.cn (A.X.); tianhuafeng@th.btbu.edu.cn (H.T.); 2 Systems Engineering Institute, Academy of Military Science, People’s Liberation Army, Beijing 100010, China; zhanhuigeng@163.com

**Keywords:** electrospinning, polysaccharide, pullulan, xanthan gum, fast dissolving oral films

## Abstract

In this study, polysaccharide-based nanofibrous fast dissolving oral films (FDOFs) were developed using pullulan (PUL) and xanthan gum (XG) via electrospinning. The edible, continuous, and bead-free nanofibers with average diameters ranging from 181.17 nm to 260.84 nm were prepared. The morphological, thermal, mechanical, and water-soluble properties of the nanofibrous FDOFs were characterized. For prospective future applications of the developed PUL/XG FDOFs, a model nutrient of vitamin C (VC) was encapsulated into the FDOFs. The success of VC encapsulation was confirmed by Fourier transform infrared spectroscopy. The encapsulation efficiency of VC was above 85% by ultraviolet-visible spectrophotometer. The amorphous structure of PUL/XG in the nanofibers film was demonstrated by X-ray diffractometer. In addition, the edible FDOFs could dissolve in water within 3 s. The nanofibers film we prepared could be used as nutrient or drug carriers and edible packaging film.

## 1. Introduction

Fast dissolving oral film (FDOF), a thin film containing dispensed active pharmaceutical ingredients, can rapidly disintegrate or dissolve in the saliva prior to swallowing, when applied to the tongue or oral mucosa of a patient [1,2]. Compared with conventional solid dose forms, such as tablets and capsules, FDOF has the advantages of increasing patient compliance, for example, allowing geriatric, pediatric, or bedridden patients who have difficulties in chewing or swallowing to take medicines. FDOF can also preserve water, allow rapid medicine release, and improve bioavailability by avoiding first-pass metabolism, etc. [1,2]. In recent years, FDOF has evolved from the confection and oral care markets to become a novel form for delivering nutrients such as vitamins in the personal care products market [3]. A variety of techniques have been developed to prepare FDOF, such as electrospinning, solvent casting, semisolid casting, hot melt extrusion, solid dispersion extrusion, and rolling [3]. Among them, electrospinning has the advantages of easy operation, low cost, and versatility [2]. Electrospinning involves an electrohydrodynamic process of drawing charged threads of polymer solutions or polymer melts to fabricate continuous fibers at a submicron or nanometer scale [4]. The performance of the electrospun nanofibrous films can be improved by properly combining or modifying the polymers and adjusting the electrospinning setup and procedure [5,6]. Compared with traditional methods, electrospinning can generate highly porous FDOF with improved fast-wetting properties and allow encapsulation of thermally unstable compounds [7,8]. Several electrospinning methods have been reported in preparing FDOF, including blend, coaxial, side-by-side, tri-axial, and layer-by-layer electrospinning [2]. Selection of the proper polymer played a key role in fabricating FDOF with the desired performance. Synthetic, semi-synthetic, and natural hydrophilic polymers, such as hydroxypropyl methylcellulose, polyvinylpyrrolidone and polyvinyl alcohol (PVA), chitosan, and PUL, have been used to provide solutions for electrospinning [2,9,10]. Among them, natural biopolymers which are biocompatible, renewable, and biodegradable have attracted great research interest [11].

Xanthan gum (XG), which contains glucose, mannose, and glucuronic acid, is an extracellular polysaccharide produced by Xanthomonas campestris [12]. XG has a high molecular weight and a polyanionic character due to the presence of pyruvic and acetic residues. XG exhibits a helical structure as a single or double helix in its native state and translates into a double-helix ordering state due to the intra- and intermolecular interactions at high salt and polymer concentrations [12,13]. The pyruvyl content plays an important role in affecting the viscosity of XG aqueous solution, i.e., higher pyruvyl content yielded high viscosity and promoted gel behavior [14]. XG has properties of non-Newtonian behavior, low sensitivity of viscosity to salinity changes, high viscosity at low concentrations, good stability of viscosity over a wide range of salt, pH, and temperature, resistance to mechanical and enzymic degradation by amylases and many cellulases, and good solubility in both hot and cold water [13,15]. XG has wide industrial applications as a thickener and emulsion stabilizer [14]. XG nanofibers have been prepared by electrospinning in formic acid and in the absence of other polymers [12].

Pullulan (PUL) is an extracellular polysaccharide produced by yeasts [16]. It is nontoxic, biocompatible, nonmutagenic, nonimmunogenic, water-soluble, biodegradable, edible, and inert to mammalian amylases [16,17]. It also has good fiber-forming properties and good stability in the presence of most metals and changes in temperature and pH [16,17]. PUL has been widely used in drug delivery (injectable and oral), gene delivery, wound dressings, tissue engineering, cosmetics, and medical imaging [17]. PUL can be used alone or mixed with other polymers to produce nanofibers by electrospinning. For example, PUL was blended with protein including pea protein isolate, amaranth protein isolate, and whey, to prepare electrospun PUL-protein nanofibers [18,19,20]. PUL was incorporated into jelly fig polysaccharide to produce nanofibers via electrospinning [21]. The blended nanofibers had fast dissolution in water and great potential for oral drug delivery [21]. PUL was blended with chitosan to prepare FDOF by electrospinning [2]. PUL was also blended with tetracyclin-cyclodextrin to prepare FDOF by electrospinning [22].

In this work, fully polysaccharide-derived nanofibrous FDOFs were developed using PUL and XG via electrospinning. To the best of our knowledge, the electrospinning of PUL and XG composites has been rarely reported. For prospective future applications of the developed PUL/XG FDOFs, a model nutrient of vitamin C (VC) was encapsulated into them for further study.

## 2. Materials and Methods

### 2.1. Materials

Pullulan (PUL) was purchased from Kangnaxin (Weifang, China), xanthan gum (XG) was purchased from Meihua (Wujiaqu, China), vitamin C (VC) was bought from Shiyao (Shijiazhuang, China), and deionized water was prepared in the laboratory.

### 2.2. Preparation of Solution for Electrospinning

PUL (20 wt.%) and XG (1.5 wt.%) were respectively dissolved in deionized water to prepare a polymer solution under magnetic stirring at 600 rpm overnight. Then, PUL and XG solutions were mixed at a weight ratio of 10:0, 9:1, 8:2, 7:3, 6:4, and 5:5, and magnetically stirred at 600 rpm for 1 h to obtain the solution named P10X0, P9X1, P8X2, P7X3, P6X4, and P5X5, respectively. In addition, VC (5 wt.%, 10 wt.%, and 15 wt.% of dry weight of polymers) was added to P9X1 solution to obtain the solution named P9X1V5, P9X1V10, and P9X1V15, respectively.

### 2.3. Preparation of Nanofibrous Film via Electrospinning

The polymer solution was loaded into a 10 mL syringe with a 22 gauge blue blunt needle and placed on the electrospinning machine (Yuyue, Shanghai, China). The voltage was set at 20 kV, the injection speed was 1 mL/h, and the distance between the needle and roller was 15.5 cm during the process of electrospinning. Nanofibers prepared by electrospinning at a speed of 400 rpm were collected by a roller, which was covered with a piece of silicone oil paper. The electrospun nanofibers named P10X0, P9X1, P8X2, P7X3, P6X4, and P5X5 were prepared by the solution of P10X0, P9X1, P8X2, P7X3, P6X4, and P5X5, respectively. In addition, the electrospun nanofibers produced by the solution of P9X1 which contained 5 wt.%, 10 wt.%, and 15 wt.% VC were named P9X1V5, P9X1V10, and P9X1V15, respectively.

### 2.4. Characterization of Solution

The viscosity of the solution was measured by a viscometer (LICHEN, Shanghai, China), using the no. 3 rotating shaft at 60 rpm, and the conductivity of the solution was measured by a conductivity meter (Yueping, Shanghai, China). The results were expressed as mean ± standard deviation from triple measurements.

### 2.5. Morphological Characterization of Nanofibrous Films

The film samples were first coated with gold and characterized using a scanning electron microscope (SEM) (FEI Quanta 200 F, FEI, Hillsboro, OR, USA) at 10 kV voltage. At least 50 fibers of each film sample were randomly selected for the average diameter measurement by an Image J 1.53 software (National Institute of Health, Bethesda, MD, USA), and the result was expressed as mean ± standard deviation.

### 2.6. Encapsulation Efficiency of VC

First, VC aqueous solutions at concentrations of 2, 4, 6, 8, 10, 12, 14, and 16 mg/L were prepared, and then the calibration curve of VC was obtained by plotting the absorbance data of the solutions at 265 nm, measured by an ultraviolet and visible spectrophotometer (Cary 60, Agilent, Santa Clara, CA, USA). To measure the actual VC content of the nanofibrous films, the film sample was dissolved in deionized water, and the absorbance of the solution at 265 nm was measured. The VC content was calculated by the calibration curve.


$$\mathrm{Encapsulation}\;\mathrm{efficiency}\;(\%)=\frac{\mathrm{actual}\;\mathrm{VC}\;\mathrm{content}\;\mathrm{in}\;\mathrm{nanofibers}}{\mathrm{theoretical}\;\mathrm{VC}\;\mathrm{content}\;\mathrm{in}\;\mathrm{nanofibers}\;}\times 100\%$$

### 2.7. Fourier Transform Infrared (FTIR) Spectroscopy

The absorbance of the nanofibrous film was measured at wave numbers ranging from 4000 to 400 cm^−1^ with a resolution of 4 cm^−1^ by a Fourier transform infrared spectrometer (NICOLET iN10 MX, Thermo Scientific, Waltham, MA, USA).

### 2.8. Thermogravimetric Analysis (TGA) of Nanofibrous Films

About 5 mg of the film sample was put in a crucible and heated to 600 °C at 10 °C/min using a thermogravimetric infrared spectrometer (Q500, TA Instruments, New Castle, DE, USA). The thermal decomposition curves of PUL, XG, VC power, and each kind of nanofibers film were measured.

### 2.9. X-ray Diffraction (XRD) Analysis

PUL, XG powder, and each kind of nanofibers films were placed on an X-ray diffractometer (Ultima IV, Rigaku, Tokyo, Japan), and their XRD curves were measured at 2θ degree ranging from 10° to 90° and a scanning rate of 5°/min.

### 2.10. Mechanical Characterization of Nanofibrous Films

The tensile strength, tensile elongation at break, and elastic modulus of the film samples (30 mm × 10 mm) were measured using a universal tensile testing machine (Xinsansi, Shenzhen, China) at a speed of 5 mm/min. The results were expressed as mean ± standard deviation from three measurements.

### 2.11. Water Dissolution Testing of Nanofibrous Films

The nanofibrous film (1.5 cm × 1.5 cm) was dissolved in deionized water, and the dissolution process was recorded by a camera.

## 3. Results and Discussion

### 3.1. Characterization of Solutions

The viscosity and conductivity of the solution played an important role in affecting the electrospinning process and the nanofibers’ morphology. Usually, good nanofibers can be prepared with sufficient viscosity and conductivity of the solution [23,24]. As shown in Table 1, the viscosity and conductivity of the solution of P10X0 were 1707 ± 0.82 mPa·s and 5220 ± 8.16 μs/cm, respectively. But with the decrease in PUL proportion and the increase in XG proportion, the viscosity and conductivity of the solution gradually decreased. Similarly, it was reported that with the decrease in PUL and the increase in whey protein, the viscosity of the solution decreased gradually [20]. The addition of VC slightly decreased the viscosity of the P9X1 solution, and as the concentration of VC increased, the viscosity of the solution increased. At the same time, the conductivity of the P9X1 solution increased with VC loading because of its ionization in water.

### 3.2. Morphological Characterization of Nanofibrous Films

**Figure 1:**
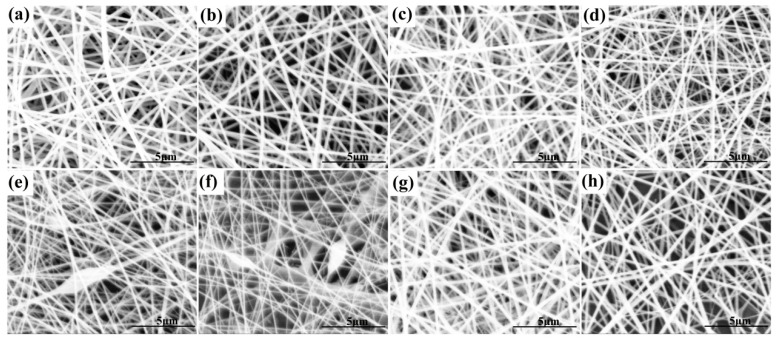
Electrospun PUL/XG hybrid nanofibers (**a**) P10X0 (**b**) P9X1, at 20 kV (**c**) P8X2 (**d**) P7X3 (**e**) P6X4 (**f**) P5X5 (**g**) P9X1, at 18 kV (**h**) P9X1, at 16 kV.

As shown in Table 2, with the decrease in PUL proportion and the increase in XG proportion, the average diameter of the prepared nanofibers gradually decreased, which was consistent with the gradual decrease in the viscosity of the solution (Table 1). Similarly, the average diameter of electrospun nanofibers of PUL/gelatin reduced from 282 nm to 160 nm with the decrease in PUL proportion and the increase in gelatin proportion [25]. As shown in Figure 1, the micromorphology of the nanofibers of P10X0, P9X1, P8X2, and P7X3 was continuous, smooth, and bead-free. However, with the decrease in PUL and the increase in XG, the nanofibers of P6X4 and P5X5 began to show bead defects. In addition, at 16, 18, and 20 KV, the average diameter of P9X1 nanofibers was 225.85 ± 51.45 nm, 206.63 ± 38.95 nm, and 181.17 ± 21.03 nm, respectively. The average diameter of P9X1 nanofibers decreased with the increase in voltage because the electrospinning was driven by the electric field force. The higher the electrospinning voltage was, the greater the driving force was, and the smaller the average diameter of the obtained nanofibers was. A similar study showed that the average diameter of electrospun nanofibers produced by whey protein/PUL (30:70) decreased from 217 ± 33 nm to 195 ± 35 nm when the voltage was changed from 18 kV to 22 kV [20].

**Figure 2:**
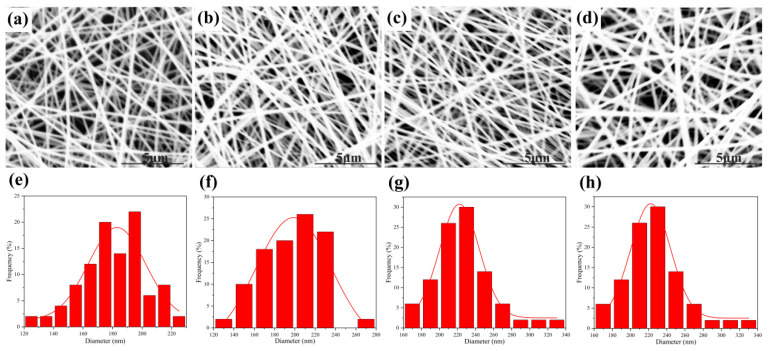
SEM of the nanofibers of (**a**) P9X1, at 20kV (**b**) P9X1V5 (**c**) P9X1V10 (**d**) P9X1V15 and the diameter distribution of the nanofibers of (**e**) P9X1, at 20kV (**f**) P9X1V5 (**g**) P9X1V10 (**h**) P9X1V15.

The effect of the addition of VC on the morphology of nanofibrous films was studied. As shown in Figure 2, P9X1, P9X1V5, P9X1V10, and P9X1V15 nanofibers had continuous, smooth, and bead-free fibers with diameters ranging from 181.17 nm to 260.84 nm. The average diameter of nanofibers increased with the increase in VC content, which could be attributed to the increase in hydrogen bonding between PUL, XG, and VC. Meanwhile, the diameter distribution was widened with the increase in VC loading.

### 3.3. Encapsulation Efficiency of VC

**Figure 3:**
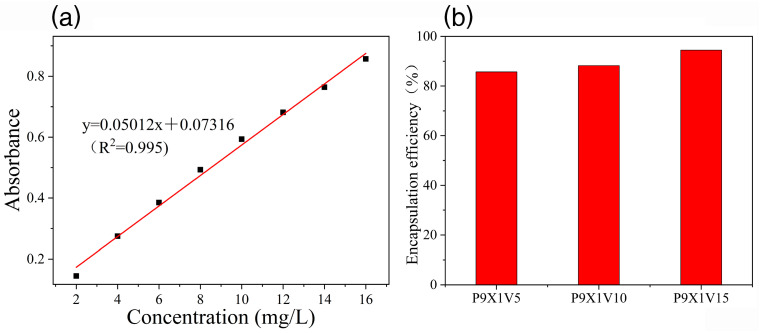
(**a**) Calibration curve of VC (**b**) Encapsulation efficiency of VC in nanofibers.

To investigate the encapsulation efficiency (EE) of VC in the nanofibers, a calibration curve was plotted to measure its concentration (Figure 3a). The EE of VC in P9X1V5, P9X1V10, and P9X1V15 nanofibers was 85.71%, 88.23%, and 94.48%, respectively (Figure 3b). With the increase in the addition of VC, its EE increased because the hydrogen bonds among PUL, XG, and VC increased. In addition, oxidation of VC during the electrospinning process might have caused a loss of EE. Similarly, with the addition of tea polyphenols ranging from 5% and 10% to 15%, its EE in the electrospun nanofibers of PVA and high amylose corn starch increased gradually [26].

### 3.4. FTIR Analysis

**Figure 4:**
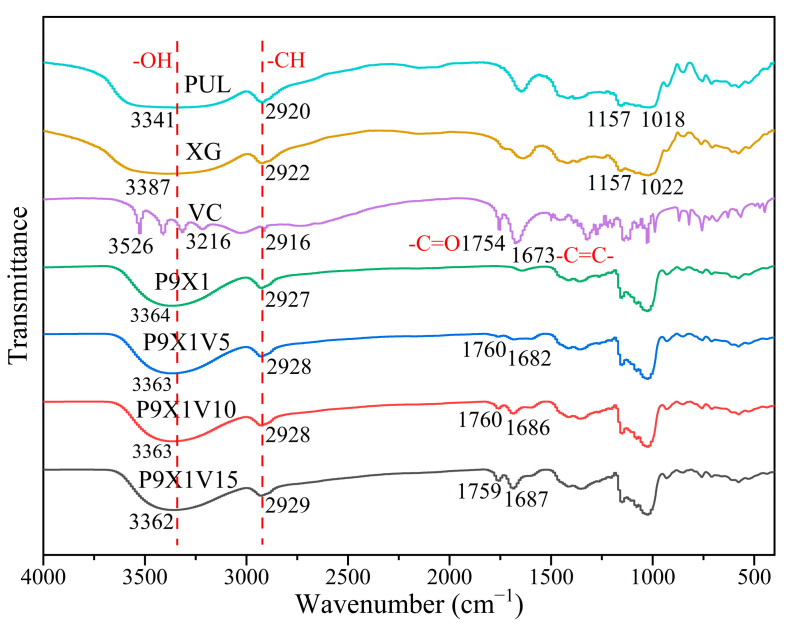
FTIR curves of PUL, XG, and VC powder and nanofibrous films of P9X1, P9X1V5, P9X1V10, and P9X1V15.

As shown in Figure 4, in the spectra of PUL, the broad peak at 3341 cm^−1^ was attributed to -OH stretching vibration, the peak at 2920 cm^−1^ was corresponded to -CH stretching vibration, and the peaks at 1157 cm^−1^ and 1018 cm^−1^ were assigned to -C-O- stretching vibration. Similarly, in the spectra of XG, the broad peak at 3387 cm^−1^ was assigned to -OH stretching vibration, the peak at 2922 cm^−1^ was due to -CH stretching vibration, and the peaks at 1157 cm^−1^ and 1022 cm^−1^ were corresponded to -C-O- stretching vibration. In the spectra of VC, the peaks at 3526 cm^−1^ and 3216 cm^−1^ were due to -OH stretching vibration, the peak at 2916 cm^−1^ was due to CH stretching vibration, the peak at 1754 cm^−1^ was due to the stretching vibration of -C=O, the peak at 1673 cm^−1^ was assigned to the stretching vibration of -C=C-, and the peak at nearby 1000 cm^−1^ was attributed to -C-O- stretching vibration. Compared with PUL and XG powder, the hydrogen bonds of P9X1 nanofibers shifted to 3364 cm^−1^, meaning that there was a formation of new hydrogen bonds between them. In comparison to P9X1 nanofibers, the hydrogen bonds of P9X1V5, P9X1V10, and P9X1V15 nanofibers were slightly shifted, which meant that there were new hydrogen bonds interactions among them. With the increase in VC content, its characteristic peak at 1754 cm^−1^ moved slightly, and another characteristic peak at 1673 cm^−1^ gradually moved to 1687 cm^−1^, which meant the strengthening of interactions between the VC and polymers. Moreover, with the increment in VC, its characteristic peak intensity at 1754 cm^−1^ and 1673 cm^−1^ enhanced gradually, which meant the success of its encapsulation in nanofibers.

### 3.5. Thermal Stability

**Figure 5:**
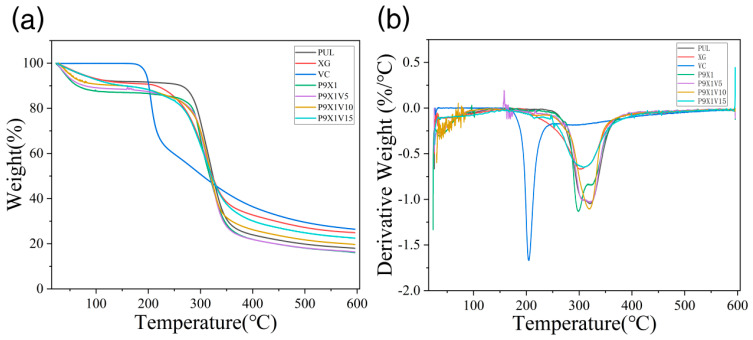
(**a**) TGA diagrams of PUL, XG, and VC powder, and P9X1, P9X1V5, P9X1V10, and P9X1V15 nanofibrous films (**b**) Their derivative thermogravimetric curves.

Polysaccharides and proteins could be used as wall materials for the encapsulation of environmentally sensitive substances such as tannic acid (polyphenol), quercetin (flavonoid), curcumin (polyphenol), vitamin B-6 (pyridoxine), etc. [27,28,29,30]. As shown in Figure 5, in the curve of PUL power, the stage before 100 °C was associated with water evaporation, and the nanofibers weight between 100 °C and 250 °C was almost unchanged. PUL began to break down between 250 °C and 360 °C, and the stage beyond 360 °C was related to its further carbonization. In the curve of XG powder, the stage before 100 °C was associated with water evaporation, and the nanofibers weight was almost unchanged between 100 °C and 200 °C. XG began to degrade between 200 °C and 360 °C, and the stage beyond 360 °C was related to the further carbonization of XG. The free VC went through a three-stage thermal degradation. The weight of VC was almost unchanged before 170 °C, but it began to break down between 170 °C and 230 °C and become further charred at the stage beyond 230 °C. The curves of nanofibrous films of P9X1, P9X1V5, P9X1V10, and P9X1V15 were similar to that of PUL. Compared to free VC, the decomposition temperature of the VC encapsulated in nanofibers increased, indicating that the nanofibers improved the decomposition temperature of VC. Similarly, compared with free thymol, when encapsulated by the electrospun nanofibers prepared by PUL and carboxymethyl starch, it had a higher thermal decomposition temperature [31]. It was also shown that there was a higher thermal decomposition temperature for eugenol encapsulated by the electrospun nanofibers of PUL and eugenol-γ-cyclodextrin compared with unencapsulated eugenol [32].

### 3.6. XRD Analysis

**Figure 6:**
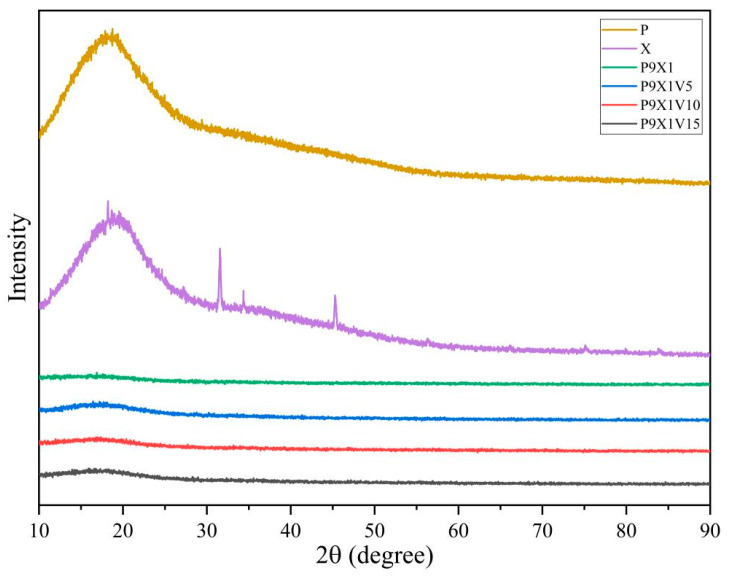
XRD curves of PUL and XG powder, and P9X1, P9X1V5, P9X1V10, and P9X1V15 nanofibrous films.

As shown in Figure 6, in the curve of PUL power, there was a broad diffraction peak at around 18.7°, indicating its weak crystalline structure. It was similar to the report that a broad peak of electrospun nanofibers of PUL was observed at 19.4° [33]. In the curve of XG power, a broad diffraction peak at 19.1° was related to its weak crystalline structure. Compared with PUL and XG powder, P9X1 nanofibers had no diffraction peak, implying that PUL and XG became amorphous structures after the change from powder to nanofibers by electrospinning. After the addition of VC, there was no diffraction in the XRD curves of the nanofibers, same as that of P9X1 nanofibers, indicating an amorphous structure in the nanofibers. Their crystallinity was weakened by the electrospinning technology. Similarly, the characteristic peaks of PUL and ethyl cellulose were weakened or almost disappeared, indicating that crystallization of the nanofibers was hindered, and more amorphous structures were formed during electrospinning [34].

### 3.7. Mechanical Properties of Nanofibrous Films

**Figure 7:**
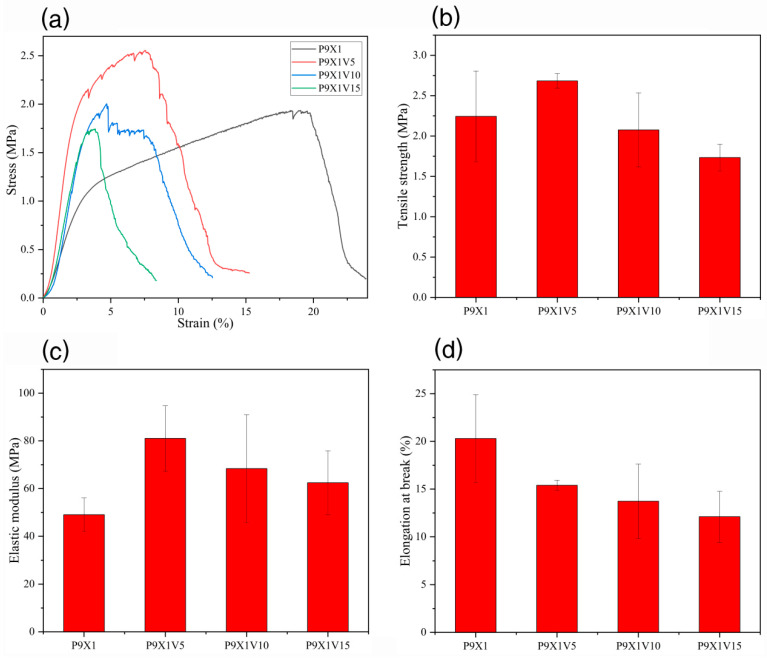
(**a**) stress-strain (**b**) tensile strength (**c**) elastic modulus (**d**) tensile elongation at break of P9X1, P9X1V5, P9X1V10, and P9X1V15 nanofibrous films.

As shown in Figure 7, the tensile strength of P9X1, P9X1V5, P9X1V10, and P9X1V15 nanofibers film was 2.24 ± 0.56 MPa, 2.68 ± 0.09 MPa, 2.08 ± 0.46 MPa, and 1.73 ± 0.17 MPa, and their elastic modulus was 49.03 ± 7.03 MPa, 81.03 ± 13.70 MPa, 68.38 ± 22.59 MPa, and 62.43 ± 13.38 MPa, respectively. Compared with P9X1 nanofibers film, the tensile strength and elastic modulus of the nanofibers film were increased after adding 5 wt.% VC. But with the further increase in VC content, the tensile strength and elastic modulus of nanofibers film gradually decreased. These results implied that adding appropriate amount of VC could increase the strength of the nanofibers film while adding more amount of it might decrease the strength of the nanofibers film. The elongation at break of P9X1, P9X1V5, P9X1V10, and P9X1V15 nanofibers film was (20.30 ± 4.60)%, (15.40 ± 0.53)%, (13.73 ± 3.89)%, and (12.11 ± 2.67)%, respectively. The addition of VC weakened the flexibility of the nanofibers film, resulting in a decrease in their tensile elongation at break.

### 3.8. Water Dissolution Properties of Nanofibrous Films

**Figure 8:**
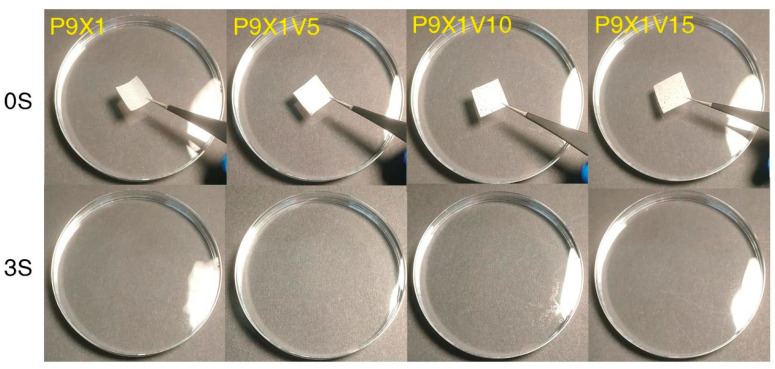
The pictures of dissolution of nanofibrous films taken at 0 s and 3 s after they were submerged in water from the Videos S1.

As shown in Figure 8, P9X1, P9X1V5, P9X1V10, and P9X1V15 nanofibrous films were fully dissolved in water within 3 s. The fast water dissolution was attributed to the hydrophilic properties of the two polysaccharides and the nanoscale porosity and high surface area of the electrospun nanofibers that can be observed from the figures of SEM (Figure 2). This was convenient for children, the elderly, and people with oral diseases because they could eat the nanofibrous FDOFs without the use of water. Similarly, they could be broken down within 2 s in simulated artificial saliva for the electrospun nanofibers produced by PUL/hydroxypropyl cyclodextrin/tetracycline [22]. It was reported that the nanofibers film of gelatin/spirulina protein concentrate prepared by electrospinning could dissolve in water within 2 s [35].

## 4. Conclusions

In this study, edible nanofibrous FDOFs of natural macromolecules PUL/XG were successfully prepared by electrospinning. The prepared nanofibers were continuous, smooth, and bead-free. They were used as carriers of VC for further study, and TGA curves displayed that the nanofibers improved the decomposition temperature of VC after encapsulation. The water dissolution test showed that the nanofibers films could dissolve quickly within 3 s, indicating that eating them was convenient for children, the elderly, and people with oral diseases with no use of water. The edible nanofibrous FDOFs of PUL/XG by electrospinning could be potentially used in nutrient or drug carriers and edible packaging films.

## Figures and Tables

**Table 1:** Viscosity and conductivity of solutions.

Solution	Viscosity (mPa·s)	Conductivity (μs/cm)
P10X0	1707 ± 0.82	5220 ± 8.16
P9X1	1501 ± 0.82	5143 ± 4.71
P8X2	1149 ± 0.82	4750 ± 8.16
P7X3	1090 ± 0.82	4390 ± 8.16
P6X4	1069 ± 0.82	4010 ± 8.16
P5X5	1061 ± 1.25	3737 ± 9.43
P9X1V5	1280 ± 0.47	5233 ± 4.71
P9X1V10	1395 ± 0.47	5270 ± 8.16
P9X1V15	1435 ± 0.47	5337 ± 4.71

**Table 2:** The process parameters of electrospinning and average diameter of nanofibers.

Nanofibrous Film	Voltage (kV)	Injection Speed (mL/h)	Roller Speed (rpm/min)	Microscopic Topography	Average Diameter of Nanofibers (nm)
P10X0	20	1	400	smooth nanofibers	198.26 ± 18.07
P9X1	20	1	400	smooth nanofibers	181.17 ± 21.03
P9X1	18	1	400	smooth nanofibers	206.63 ± 38.95
P9X1	16	1	400	smooth nanofibers	225.85 ± 51.45
P8X2	20	1	400	smooth nanofibers	161.52 ± 22.88
P7X3	20	1	400	smooth nanofibers	138.71 ± 23.55
P6X4	20	1	400	smooth nanofibers with few beads	124.56 ± 17.78
P5X5	20	1	400	smooth nanofibers with few beads	105.17 ± 21.09
P9X1V5	20	1	400	smooth nanofibers	197.89 ± 29.92
P9X1V10	20	1	400	smooth nanofibers	224.18 ± 30.99
P9X1V15	20	1	400	smooth nanofibers	260.84 ± 39.58

## Data Availability

Data are contained within the article and Supplementary Materials.

## Supplementary Materials

The following supporting information can be downloaded at: https://www.mdpi.com/article/10.3390/ma17040861/s1, Videos S1: Videos of nanofibrous films dissolving in water.
